# Methylxanthines and Neurodegenerative Diseases: An Update

**DOI:** 10.3390/nu13030803

**Published:** 2021-02-28

**Authors:** Daniel Janitschke, Anna A. Lauer, Cornel M. Bachmann, Heike S. Grimm, Tobias Hartmann, Marcus O. W. Grimm

**Affiliations:** 1 Experimental Neurology, Saarland University, 66421 Homburg/Saar, Germany; Daniel.Janitschke@uks.eu (D.J.); Anna.Lauer@uks.eu (A.A.L.); Manuel.Bachmann@uks.eu (C.M.B.); Heike.Grimm@uks.eu (H.S.G.); Tobias.Hartmann@uks.eu (T.H.); 2 Deutsches Institut für DemenzPrävention (DIDP), Saarland University, 66421 Homburg/Saar, Germany

**Keywords:** methylxanthines, caffeine, theobromine, theophylline, pentoxifylline, propentofylline, istradefylline, Alzheimer´s disease, Parkinson´s disease, Multiple Sclerosis

## Abstract

Methylxanthines (MTX) are purine derived xanthine derivatives. Whereas naturally occurring methylxanthines like caffeine, theophylline or theobromine are widely consumed in food, several synthetic but also non-synthetic methylxanthines are used as pharmaceuticals, in particular in treating airway constrictions. Besides the well-established bronchoprotective effects, methylxanthines are also known to have anti-inflammatory and anti-oxidative properties, mediate changes in lipid homeostasis and have neuroprotective effects. Known molecular mechanisms include adenosine receptor antagonism, phosphodiesterase inhibition, effects on the cholinergic system, wnt signaling, histone deacetylase activation and gene regulation. By affecting several pathways associated with neurodegenerative diseases via different pleiotropic mechanisms and due to its moderate side effects, intake of methylxanthines have been suggested to be an interesting approach in dealing with neurodegeneration. Especially in the past years, the impact of methylxanthines in neurodegenerative diseases has been extensively studied and several new aspects have been elucidated. In this review we summarize the findings of methylxanthines linked to Alzheimer´s disease, Parkinson’s disease and Multiple Sclerosis since 2017, focusing on epidemiological and clinical studies and addressing the underlying molecular mechanisms in cell culture experiments and animal studies in order to assess the neuroprotective potential of methylxanthines in these diseases.

## 1. Introduction

This review summarizes on the latest findings of the relationship between methylxanthines and neurodegenerative diseases, especially Alzheimer’s disease (AD), Parkinson´s disease (PD) and Multiple Sclerosis (MS). These multifactorial disorders share several common histopathological hallmarks and mechanisms like neuronal cell loss linked to gliosis, misfolding and accumulation of proteins, oxidative stress and neuroinflammation. In case of AD, disease progression is strongly associated with Aβ peptide generation and aggregation, associated with pathological extracellular and intracellular filamentous deposits, hyperphosphorylated tau proteins, neuroinflammation and synaptic loss. PD is characterized by dopaminergic neurodegeneration and accumulation of α-synuclein in Lewy bodies. Concerning MS, neuroinflammatory processes leading to demyelination of neurons are in the focus of the disease.

Numerous studies have been performed to address the question of whether xanthine derivatives like caffeine and theobromine have beneficial properties in respect to the characteristic histopathological changes that occur in the above-mentioned diseases and to cognitive decline. Although the outcome of the clinical studies, especially for AD, were heterogeneous [1], some mechanisms were identified, showing how methylated xanthine derivatives could protect against neuronal damage. 

In general, in physiological concentrations, achieved for example by coffee consumption or by intake of methylxanthine containing beverages, methylxanthines act as antagonists of the adenosine receptor (AR), histone deacetylase activator or antioxidant. Affecting these pathways, xanthine derivatives are able to modulate molecular mechanisms associated with neurodegenerative diseases like accumulation of misfolded proteins, oxidative stress and neuroinflammation. Interestingly, in particular the beneficial role of adenosine receptor antagonists in the treatment of neurodegenerative diseases has become more and more apparent in the last years [2,3,4,5].

In supraphysiological concentrations, which can be reached for example, by intake of methylxanthines as pharmaceuticals, additional mechanisms like inhibition of phosphodiesterases and high-affinity ATP-dependent cyclic nucleotide transporters are reported, also contributing to neuroprotective properties of methylxanthines [6]. Besides the above-mentioned properties of xanthine derivatives, further mechanisms were suggested or excluded in the last years and are summarized in detail below together with new clinical trials addressing these issues (also see figures and tables).

## 2. Alzheimer’s Disease

### 2.1. Epidemiological and Clinical Studies

In the past four years, several new clinical and epidemiological studies were published examining the influence of caffeine or other methylxanthines on cognitive performance in respect to AD, see Table 1.

Jee Wook Kim and colleagues analyzed the relationship between lifetime coffee intake and AD biomarkers in the human brain using neuroimaging techniques like positron emission tomography (PET), magnetic resonance imaging scans and clinical assessment. The study included 411 participants with an inclusion criterion for patients already suffering from mild cognitive impairment (MCI) but without a diagnosis of dementia. Study participants were divided into high and low/no coffee consumer: 269 participants, consuming no coffee or <2 cups/day, and 142 participants stated to drink two or more than two cups per day, reflecting higher coffee intake. The authors reported that a higher lifetime coffee intake was significantly associated with a reduced pathological cerebral amyloid deposition and could therefore be linked to a lowered risk for AD or cognitive decline even after controlling for potential cofounders. Besides the association of coffee intake with reduced amyloid deposition, no correlation between coffee intake and AD associated glucose hypometabolism, atrophy or cortical thickness and cerebral white matter hyperintensities were detected [7]. This study is in line with older results obtained by transgenic mouse models or animals revealing a decreased Aβ concentration [8,9]. Interestingly, this study also revealed that the effect was more pronounced in long-term coffee drinkers suggesting that the positive effects of higher coffee intake against Aβ pathology involve more chronic effects which are associated with a regular long-term exposure rather than a short-term effect of caffeine. These results might help to explain why some previous studies including participants with shorter caffeine exposure showed weak or no correlation of caffeine with cognitive decline or amyloid burden, emphasizing that not only the caffeine concentration but also duration of exposure plays a critical role for the beneficial effects of methylxanthines in AD.

In this context a new meta-analysis of eight observational prospective studies from Larsson and Orsini should be noticed. No statistically significant association between coffee consumption and risk of dementia were detected by the authors. The 95% confidence interval of dementia for one cup coffee/day was 0.92, two cups/day 0.90, three cups/day 0.93, four cups/day 1.01 and five cups/day 1.11. In line with the authors, it has to be mentioned that especially over a long time, coffee consumption may vary and is hard to be exactly estimated. Moreover, three studies showing effects of coffee consumption on AD risk were not included because they used different quantitative categories of coffee consumption [10,11,12,13]. Another interesting aspect is revealed by a recent study from Iranpour and colleagues analyzing data from the National Health and Nutrition Examination Survey (NHANES) including 1440 participants older than 60 years [14]. By applying different cognitive tests, the authors reported a weak positive relation of high caffeine intake with cognitive function. Importantly the correlation was stronger amongst males than females emphasizing a potential need of subgroup-analysis besides combining studies in meta-analysis to obtain large but probably even more heterogeneous cohorts. In summary, in respect to dose dependency, a correlation between coffee consumption and AD risk is still controversial and can currently not be answered without uncertainty in absence of further studies.

#### 2.1.1. Are the Positive Effects due to Caffeine or Other Compounds in Coffee?

Besides caffeine, coffee contains several biological compounds known to be biologically active—such as phenylindanes or molecules produced during roasting of coffee beans [15,16]. Therefore, it is difficult to answer the question of whether the potential positive observed effects are due to caffeine or other substances. Addressing exactly this important question, Xue Dong and colleagues analyzed in their study the association of coffee, caffeinated coffee, decaffeinated coffee and caffeine intake from coffee with cognitive performance. CERAD (Consortium to Establish a Registry for Alzheimer´s Disease) test and DSST (Digit Symbol Substitution Test) were performed with over 2500 participants from the NHANES aged 60 years or older without any diagnosis of AD. Significant associations with cognitive performance were reported for coffee, caffeinated coffee and caffeine from coffee, but not for decaffeinated coffee [17], underlining the important role of the methylxanthine caffeine in coffee. However, as older studies could also show beneficial effects of other compounds in coffee—besides caffeine—this result should emphasize the impact of caffeine but does not rule out that other substances might contribute, especially in combination with caffeine, to a protective effect as well [18,19]. 

#### 2.1.2. The Effect of Other Methylxanthines on Alzheimer’s Disease

Besides caffeine, several other methylxanthines are known that have potential protective effects in respect to their molecular mechanism. However, very little is known about their potential protective function in respect to AD in clinical studies or data obtained from human samples. De Leeuw investigated the association of different nutritional biomarkers with clinical progression in patients with cognitive decline. Theobromine revealed to be increased in a subgroup of patients with lower Aβ42 level but also higher total and phosphorylated tau level. In addition, high theobromine levels were found to be associated with cognitive decline in mild cognitive impairment (MCI) patients [20]. At first glance, these results might point towards a negative impact of methylxanthines in disease progression. However, it has to be taken into consideration, that caffeine is metabolized in the liver to theobromine. A faster or altered metabolization rate of caffeine might result in an increase in theobromine but in a decreased caffeine level. Theobromine compared to caffeine has been shown to have a lower anti-amyloidogenic potential [21]. Therefore, the association of increased theobromine with cognitive decline might be influenced by the faster degradation of caffeine resulting in lower caffeine levels. However, as caffeine is not studied in this paper this potential explanation is speculative. Nevertheless, in line with this argumentation, a recent article from Mullins and colleagues discussed if genome-wide single nucleotide polymorphism (SNP) data can help to predict the individual response to dietary interventions. The *CYP1A2* gene encodingcytochrome P450 1A2, which is responsible for approximately 95% of caffeine metabolization and thirteen SNPs are associated with this gene. One of these SNPs (rs762551) influences the sensitivity for caffeine and how fast it gets metabolized [22]. Unfortunately, it is unknown whether this SNP is associated with AD, but undoubtedly—this SNP in the *CYP1A2* gene having an impact on caffeine intake might explain the heterogeneous results of clinical studies and should be taken into consideration for further clinical trials, since SNP data are nowadays quickly and inexpensively acquired. Therefore, in clinical studies *CYP1A2* variants have to be considered as an additional important factor highly affecting the pharmacokinetic of methylxanthines. Importantly, the pharmacokinetic of methylxanthines can be further influenced by lifestyle or dietary habits. For example, caffeine consumption combined with smoking cessation has been reported to be associated with more than two times increased caffeine plasma levels that could even induce caffeine toxicity symptoms [23]. In summary, the lack of knowledge of the methylxanthine plasma levels or the lifestyle habits which interfere with the pharmacokinetics of methylxanthines, and the additional analysis of *CYP1A2* variants, makes it hard to estimate the potency of methylxanthines in treating or preventing neurodegenerative diseases and should be considered as a caveat in interpreting previous studies.

Moreover, synthetic methylxanthines are an interesting substance class which might be suitable to treat or prevent AD, because of their capability to act as phosphodiesterase inhibitors. A recent systematic review of clinical trials, epidemiology and meta-analyses reported propentofylline as the only phosphodiesterase inhibitor having completed efficacy-testing clinical trials showing improvement of cognition and dementia severity in mild-to-moderate AD patients. Propentofylline was found to be the most effective inhibitor out of phosphodiesterase-inhibiting xanthine derivatives by inhibiting several phosphodiesterase isoforms, especially phosphodiesterase 2 and 4. The authors therefore suggest a co-treatment of propentofylline with the phosphodiesterase 5 inhibitor sildenafil for prevention or treatment of AD [6]. Based on these data further clinical trials analyzing the effect of this synthetic methylated xanthine on AD symptoms and severity should be performed.

It has to be considered that clinical studies could have limitations like un-representative sample populations, implications of confounds or personal motivation of the participants for self-reporting of their coffee consumption. Other interesting points are the individual caffeine sensitivity and the above mentioned pharmacokinetic of methylxanthines.

### 2.2. Animal Studies/Molecular Pathways

Concerning the uptake of caffeine, an animal study from Liang Jin and colleagues shows that intestinal permeability and oral absorption of caffeine were not affected in a mouse model of familial AD. They reported that plasma caffeine concentration as well as total brain exposure did not differ between wildtype and APP/PS1 mice after oral administration. APP/PS1 mice contain human transgenes for both APP bearing the Swedish mutation and PSEN1 containing the L166P mutation, therefore representing a transgenic mouse model that overproduces Aβ and that is often used to study the neuropathologic mechanisms of AD as well as the therapeutic effects of drugs on AD [26]. In line with this, the authors demonstrated in a previous study, that the abundance of intestinal and hepatic Cyp1a2 was not different in APP/PS1 mice in comparison to wildtype mice [27]. These data suggest caffeine as potential therapy for AD, since its uptake is not impaired in patients suffering from this neurodegenerative disease. Another animal study, performed by Zappettini and colleagues, used a mouse model of AD-like Tau pathology (THY-Tau22 transgenic mice) for investigation of the long-term consequences of early life exposure to caffeine during pregnancy. Their findings suggest that Tau pathology related pathological traits appear earlier in the offspring of caffeine exposed mice and therefore suggest caffeine exposure during pregnancy as a risk factor for early onset AD like pathology [28].

In addition to caffeine, the neuroprotective effects of its metabolite theobromine were also analyzed in animal studies. Yoneda and colleagues reported that orally administered theobromine (0.05% for 30 days) is detectable in plasma and cerebral cortex in wild type mice. (The utilized mouse strain here was C57BL/6NCr mice, the nomenclature of these mice is due solely to their origin, since after establishing the substrain C57BL/6 at The Jackson Laboratory the sublines C57BL/6N and C57BL/6J were separated and Cr stands for Charles River because this company acquired their breeding colonies in 1974 [29]). It can act as phosphodiesterase inhibitor in the brain and enhances cAMP/CREB/BDNF pathways in a way that supports cell survival and neuronal functions. Moreover, the theobromine-fed mice showed better performances on a three-lever motor learning task [30]. These findings suggest a beneficial influence of cacao products on learning and memory.

In the case of the adenosine A2 receptor (A2AR) antagonist istradefylline, a pharmaceutic that is approved for PD in Japan (for detailed information see later), an animal study performed by Orr and colleagues investigated the ability of this reagent to enhance cognitive functions in aging mice with AD-like amyloid plaque pathology. The authors reported increased spatial memory and habituation in APP transgenic mice treated with low doses of istradefylline (≤10 mg/kg/day) and underline the importance of further investigations of this adenosine receptor antagonist as potential therapeutic approach for AD or other neurodegenerative diseases besides PD [31]. In this context, a recent in vitro study from Franco and colleagues suggests that antagonists of A2AR affects the function of N-methyl D-aspartate ionotropic glutamate receptors (NMDAR), since A2AR activation leads to higher NMDAR functionality in neurons [32].

In the past years several experimental studies were performed to elucidate the molecular mechanisms that underlie the observed beneficial effects of methylxanthines like caffeine in AD. Gastaldo and colleagues examined in their study if food ingredients (among others caffeine) are able to affect Aβ peptide aggregation in AD through an indirect, membrane-mediated pathway, since membranes are known to play a crucial role in early stages of peptide aggregation. The authors used synthetic brain membranes to analyze the influence of caffeine on size and volume fraction of aggregates consisting of cross-β sheets of the membrane active fragment Aβ_25-35_. Caffeine was reported to spontaneously partition into the membranes in the first 150 ns of the molecular dynamics simulation and found to mainly position in the head-tail interface of the membranes—and some also temporarily inside the hydrophobic core. The Aβ_25-35_ peptides were found via microscopy to form pronounced amyloid fibrils, located on the top of the membranes in the presence of caffeine. Moreover, using X-ray diffraction they found that caffeine leads to membrane thickening and to a decrease in membrane fluidity and in presence of the Aβ_25-35_ peptides an increase in local membrane curvature was reported, which is likely induced by the formation of extracellular Aβ aggregates and fibrils. Additionally, they found in their UV-visible spectroscopy studies using thioflavin T, a significant increase in the fluorescent signal of β-sheets at 420 nm after addition of caffeine [33]. Similar results regarding the influence of caffeine on membranes were obtained in one of the authors’ earlier studies [34]. In respect to caffeine and Aβ peptides, Gupta and colleagues reported as a result of their molecular dynamics simulations a disorganization of cross-β structures of Aβ_17-42_ fibrils in the presence of caffeine. This destabilization effect might further inhibit the formation of aggregates [35]. In respect to Aβ homeostasis, Janitschke and colleagues examined the effects of the methylxanthines caffeine, theobromine, theophylline, pentoxifylline and propentofylline in human neuroblastoma cells. They concluded that the analyzed xanthine derivatives reduce the levels of Aβ via pleiotropic mechanisms by shifting the processing of the amyloid precursor protein from the amyloidogenic to the non-amyloidogenic pathway via influencing protein stabilities and gene expressions as well as affecting the involved secretases directly. Moreover, these methylxanthines decrease oxidative stress, levels of cholesterol and aggregation of Aβ_1-42_ in SH-SY5Y neuroblastoma cells [21].

Another pathway, by which caffeine could mediate its pharmacological activity, is the cholinergic system. In this context, Fabiani and colleagues used single channel recordings and fluorescent measurements to examine the influence of caffeine on the nicotinic acetylcholine receptor (AChR). Their results show that caffeine acts as a partial agonist and an ion channel blocker at neuronal α7 and muscle nicotinic receptors (AChR) at different concentrations and suggest this methylxanthine as a multitarget-directed drug for the treatment of AD [36]. Interestingly, Kumar and colleagues screened more than 600 molecules of natural origin for their ability to modulate acetylcholine metabolism. They found that caffeine has a comparable AChE inhibitory potential to donezepil, a commonly used drug to treat mild to moderate dementia. Moreover, caffeine shows no neurotoxicity in primary (E18) hippocampal neurons but significantly improves neuronal survival and protects from neurodegeneration [37]. 

For the efficiency of caffeine its distribution to and in the brain is highly important. It is important to mention that ATP-binding cassette (ABC) transporters in the brain can be affected and therefore might have an impact on methylxanthine distribution in the brain under pathological conditions [38]. In addition, it has been shown that caffeine is able to inhibit ABCC4 and ABCC5 with affinities of similar magnitude as for the adenosine receptor and thereby might diminish its efflux from the brain [39].

The anti-inflammatory and antioxidant potential of caffeine was analyzed by Badshah and colleagues in an animal study using lipopolysaccharide (LPS)-induced oxidative stress and neuroinflammation. LPS was identified as a Toll-like receptor 4 (TLR-4) ligand, primarily expressed in the central nervous system. Activation of TLR-4 leads to the production of proinflammatory cytokines, key mediators of the neuro-inflammatory process [40]. The authors reported upregulated expression levels of nuclear factor erythroid-2-related factor 2 and enzyme hemeoxygenase 1, two endogenous antioxidant regulators, and significantly downregulated expression levels of toll-like receptor 4, phosphor-nuclear factor kappa B and phosphor-c-jun n-terminal kinase in the LPS-injected mouse model treated with 30 mg/kg/day caffeine intraperitoneally for four weeks. Accordingly, this study was able to show—by using Western blot, immunofluorescence studies and biochemical assays—that caffeine prevents LPS-induced oxidative stress in the mouse brain and suppresses inflammatory mediators simultaneously [41]. The same authors showed in a second study using in vitro (HT-22 and BV-2 cells) and in vivo (B57BL/6N mice) models, reducing effects of caffeine in regard to reactive oxygen species, lipid peroxidation and inflammatory mediators. Cadmium-induced cognitive deficits, neuronal loss or synaptic dysfunction were rescued by caffeine in their mouse model. On a molecular level the neuroprotective effects of caffeine are exerted via regulation of nuclear factor-2 erythroid-2 and nuclear factor-κB [42]. Moreover, neuroinflammation and Aβ may cause neuronal death by activating the complement system. A potential impact of complement- and C-reactive protein (CRP)-mediated neuronal death in AD has already been discussed in previous literature. Fischer and colleagues reported increased levels of complement component 1q (C1q) in the frontal cortex of patients suffering from AD [43]. In human AD/stroke patients, a co-localization of monomeric CRP (mCRP) with Aβ plaques, tau-like fibrils and insulin receptor substrate 1 (IRS-1)/Phospho-Tau positive neurons was reported, and the authors suggested mCRP may be responsible for promoting dementia after ischemia [44]. Importantly, it has been shown that coffee consumption can reduce CRP levels and therefore reduce inflammation, which has been analyzed in a dose response meta-analysis including eleven studies and 61,047 participants. Analysis of the three studies with the largest sample sizes shows a statistically significant association between coffee and CRP levels. However, by combining all studies, no significant associations were found in a dose-response meta-analysis, indicating a further need to investigate these inconsistent associations [24]. In line with the reported slightly anti-inflammatory effects of coffee, Rodas and colleagues showed in their recent study that caffeine intake is a negative predictor of CRP. They analyzed the effect of regular caffeine intake in 244 healthy participants aged 18–55 years [25].

Recently, another additional mechanism by which caffeine might exert its potential therapeutic benefits in AD has been suggested by Zhao and colleagues [45]. In the presence of caffeine, Wnt signaling was found to be upregulated. Caffeine can bind to and inhibit the activity of notum, a hydrolase which inhibits Wnt signaling via enzymatic delipidation of Wnt ligands. Restoration of Wnt signaling is discussed to be beneficial in AD pathologies, since this pathway is essential for neuronal differentiation, development, adult neurogenesis and neural stem cell maintenance. Interestingly, only caffeine, but not its demethylated metabolites paraxanthine, theobromine or theophylline exerts this inhibitory property. Using structure analysis, the authors were able to show that caffeine binds at the enzymatic pocket and overlapped the position of notum´s natural substrate palmitoleic lipid [45]. The relatively low inhibitory potency of caffeine (IC_50_ 19 µM) indicates that in adults consuming moderate amounts of three or four cups of coffee per day, resulting in plasma concentrations of about 15 µM [46], physiological notum inhibition seems unlikely. But in the case of using caffeine as a therapeutic medicine, plasma levels of 150 µM were obtained [47]. 

Moreover, it is discussed that caffeine in physiological concentrations mediates its neuroprotective effects via its inhibitory interactions with adenosine receptors and thereby increases the concentration of adenosine, known to have neuromodulatory properties. Nabbi-Schroeter and colleagues investigated the influence of long-term caffeine consumption (30 mg/kg/day for 84 days in drinking water) on the adenosine A1 receptor (A1AR) using small animal positron emission tomography and the highly selective radioligand [18F]CPFPX in the brain of Sprague-Dawley rats. The selected dose of caffeine in this study corresponds to a human consumption of four to five cups of coffee per day, which is a common daily intake in industrialized countries. The authors reported no long-persistent upregulation of functionally available A1ARs in vivo under their experimental conditions of chronic caffeine intake, and based on this, they excluded this mechanism as a molecular basis for mediating the neuroprotective effects reported for this methylated xanthine [48]. In regard to the A1AR, Mendiola-Precoma and colleagues reported in their study—examining the effects of a cholesterol-enriched diet on cognitive processes in a rat model—that the caffeine metabolite theobromine was able to restore A1 receptor levels, which were reduced as a consequence of the diet. Moreover, theobromine showed prophylactic neuroprotection against damage to cognitive functions and levels of Aβ when 30 mg/L were added to drinking water. The authors suggest that the reducing Aβ effect could be mediated by the antioxidant and anti-inflammatory capacities of theobromine via decreased SOD1 and NFκB levels [49]. Moreover, theobromine isonucleotide analogs were synthesized and evaluated as active compounds in the inhibition of cholinesterases, since their low cytotoxicity makes them interesting as therapeutic molecules for AD [50]. Recently, Ciaramelli and colleagues reported theobromine and catechins as the chemical components of cocoa that hinder Aβ peptide aggregation and toxicity. They analyzed the contents of Lavado cocoa in respect to their anti-amyloidogenic properties using NMR spectroscopy, preparative reversed-phase chromatography, atomic force microscopy and biochemical and cell assays in a human neuroblastoma SH-SY5Y cell line, which is commonly used to elucidate underlying mechanisms in respect to AD [51].

**Figure 1:**
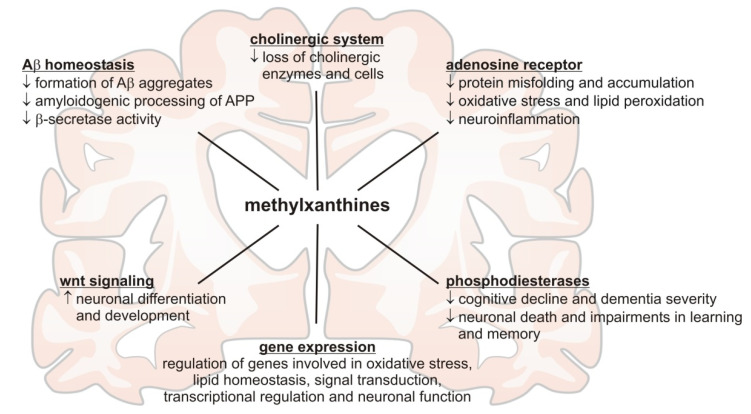
Summary of the molecular mechanisms reported in recent literature potentially mediating the beneficial effects of methylxanthines in respect to Alzheimer´s disease.

Recently, methylxanthines were reported to influence the expression of genes linked to pathways involved in processes like oxidative stress, lipid homeostasis, signal transduction, transcriptional regulation, and neuronal function, all known to be influenced in the pathophysiology of AD, by Janitschke and colleagues. In their profiling study on a human neuroblastoma cell line, they found that caffeine shows different or inverse effects on gene regulation compared to the other analyzed methylxanthines theobromine, theophylline, pentoxifylline and propentofylline [52]. In line with the outcome of the above-mentioned studies, summarized in Figure 1 and Table 2, significant differences between the individual methylxanthines were detected, further complicating a prediction of neuroprotective effects of other methylxanthines by referring to caffeine.

## 3. Parkinson’s Disease

### 3.1. Epidemiological and Clinical Studies

Parkinson´s disease (PD) is one of the most common neurodegenerative diseases characterized by a loss of dopaminergic-neurons in the substantia nigra and accumulation of misfolded α-synuclein protein aggregates in Lewy bodies. Leading symptoms of PD are bradykinesia or akinesia with rest tremor, postural instability or rigor. Additionally, patients suffering from PD show a broad range of non-motor symptoms differing in early and late-stage symptoms, e.g., obstipation and dementia. Despite an unknown etiology of PD, many risk factors such as age, male gender, environmental factors, intestinal inflammation, and genetics are described [53,54]. Besides the known risk factors it is discussed that nutrition may be associated with increased (dairy products) or decreased (phytochemicals, Omega-3 fatty acids, tea) risk or progression in PD [55]. Multiple epidemiological studies could already demonstrate an inverse association between coffee drinking and PD and thereby suggest a link to caffeine, and consequently methylxanthines, as A2A receptor antagonists [56,57,58]. Recent studies have confirmed this association (for an overview see Table 3). Bakshi et al. found a significantly lower caffeine intake, determined through questionnaire, in idiopathic PD patients compared to control group from the Harvard Biomarkers Study cohort with 369 cases of PD and 197 healthy controls [59]. In a study from Fujimaki et al., the authors investigated serum from 108 patients with PD by liquid chromatography-mass spectrometry (LC-MS) compared to 31 age-matched healthy control participants and found significantly lowered serum levels of not only caffeine, but also its downstream products like theophylline, theobromine and paraxanthine [60]. Crotty et al. demonstrated similar results in a recent metabolomics study showing a significantly lower plasma and CSF concentration of caffeine (71% lower) and its degradation products, e.g., paraxanthine and theophylline (57%, 56% lower), in PD patients versus control group measured via LC-MS. Subgroup analysis of carriers of the *LRRK2* mutation, a mutation in the leucine-rich repeat kinase 2 gene (known for an increased risk for sporadic PD) [61], revealed an even greater decrease of plasma caffeine level by 76% for PD *LRRK2* mutation carriers compared to healthy *LRRK2* carriers [62]. Ohmichi et al. found similar results for the methylxanthine analogue theophylline and confirmed a significantly lower plasma concentration in patients with PD versus an age-matched control group [63]. A recent meta-analysis by Hong et al., which included 13 studies in total, showed that for healthy individuals, caffeine consumption results in a significantly lower risk of developing PD specific symptoms (HR = 0.797, 95% CI: 0.748–0.849, *p* < 0.001, I2: ~ 15.41%, 9/13 studies). Furthermore, a significant deceleration of PD progression among early-stage PD individuals with higher caffeine consumption was found (HR = 0.834, 95% CI = 0.707–0.984, *p* = 0.03, I^2^: ~39.7%, 4/13 studies) [64]. Another interesting recent study, carried out by Maclagan et al., used a computational approach to rank 620 drugs with the ability to inhibit α-synuclein aggregation. They examined associations between the top 15 drugs in case-control validation study by using health administrative databases. Their logistic regression models found that patients exposed to methylxanthines, especially the synthetic pentoxifylline (PTX) and the natural occurring theophylline, were associated with decreased odds of occurrence of PD. Additionally longer durations of PTX administration revealed a trend towards dose-response [65]. One modified methylxanthine, istradefylline (ISD), has been used to decrease daily “off episodes”, a state where symptoms recur when the effect of L-DOPA weakens, in PD patients. ISD was approved first in Japan in May 2013 and by the FDA in the US in 2019 [66]. A meta-analysis including six studies revealed that treatment with ISD 40 mg/day decreased the duration of “off episodes” and enhanced the motor symptoms of PD patients. Similar effects could be found for 20 mg/day. ISD treatment revealed no significant effect on adverse effects. In summary the meta-analysis showed that ISD 20 mg and 40 mg both improved the unified Parkinson’s disease rating scale III (UPDRS) [67].

### 3.2. Animal Studies/Molecular Pathways

**Figure 2:**
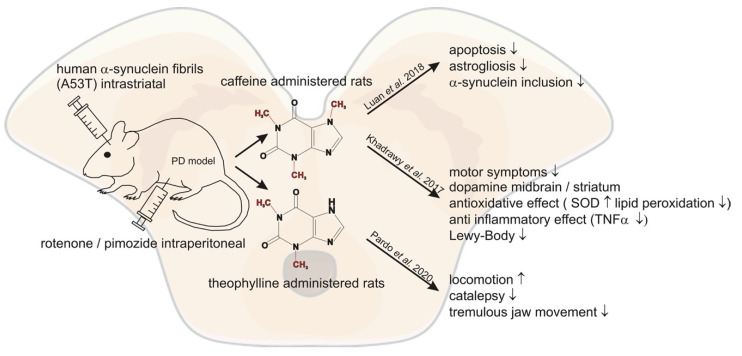
Summary of the molecular mechanisms reported in recent literature potentially mediating the beneficial effects of methylxanthines in respect to Parkinson´s disease.

Older studies have already demonstrated neuroprotective effects of caffeine in PD mouse models [68]. In line with these previous encouraging results, Luan et al. demonstrated that chronic caffeine treatment reduced the effect of intra-striatal injected human A53T α-Synuclein fibrils in mice [69]. Caffeine was administered seven days before the injection and applied for 120 days at concentrations of ~0.4–2 mg/L. They found significantly decreased inclusion of α-Synuclein in the striatum of the caffeine treated mice. Furthermore, apoptosis, microglial activation and astrogliosis were significantly reduced [69]. A similar study used 52 rats with a control group, a PD model group—generated by injecting 1.5 mg/kg rotenone intraperitoneally (i.p.) for 45 days—and two caffeine groups. One of the caffeine groups was injected with 30 mg/kg caffeine i.p. in addition to the administered rotenone for 45 days, whereas the other group was treated with caffeine after the induction of PD via rotenone. The study revealed that co-treatment and post-treatment with caffeine in the intoxicated rats enhanced the dysfunction of rotenone-induced motor symptoms by recovering dopamine levels in the midbrain and striatum. In addition, caffeine improved the antioxidant effect by reducing rotenone-induced lipid peroxidation and superoxide dismutase activity in the striatum and midbrain. Furthermore, the data revealed reduced rotenone-induced TNF-α activity in the caffeine groups indicating an anti-inflammatory effect of caffeine. Caffeine protection and treatment restored open field test parameters, forelimb hanging test and traction test to nearly control group values. Histopathological investigation revealed a degeneration of neurons and presence of Lewy bodies in the rotenone-induced PD group, which was prevented in both caffeine groups with a more prominent effect in the caffeine protected group [70]. Pardo et al. demonstrated in another animal study that the naturally occurring methylxanthine theophylline can reverse motor symptoms in rats. In this study theophylline reversed locomotion, catalepsy and tremulous jaw movement (resembling parkinsonian tremor) induced by pimozide, a D2 dopamine antagonist [71]. In conclusion, these studies show a link for caffeine and other methylxanthines acting as A2A receptor antagonists with a decreased risk for PD and modification of progression, indicating new adenosine receptor ligands might be encouraging targets in treating PD. In a study by Rohilla et al. newly synthesized xanthine derivates as selective AR antagonists were analyzed. Evaluation of the antiparkinsonian effect in the study was carried out by inducing catatonia in rats with perphenazine. Most xanthines significantly lowered the catatonic score compared to control. The most potent antiparkinsonian effect was found for the methylxanthine RB-531 (8-[3-(3-Chloropropxy)]-1,3-dipropyl-7-methylxanthine), showing a similar response as the standard treatment L-DOPA [72]. The abovementioned studies and their outcomes are summarized in Figure 2 and Table 4.

## 4. Multiple Sclerosis

### 4.1. Epidemiological and Clinical Studies

Multiple sclerosis (MS) is an autoimmune disease of the central nervous system (CNS) where the immune system impairs the myelin sheath that covers nerve fibers. Thus, communication between the CNS and the peripheral nervous system (PNS) is damaged resulting in a potentially disabling neurologic disease. The underlying cause of MS is unclear, however epidemiological studies have shown that low serum levels of Vitamin D, smoking and Epstein-Barr-Virus infections play a role in the development of the disease [73,74].

Unlike in PD or AD, a recent study from Lu et al. revealed no association between coffee consumption and the risk of MS [75] (see Table 5). 

### 4.2. Animal Studies/Molecular Pathways

**Figure 3:**
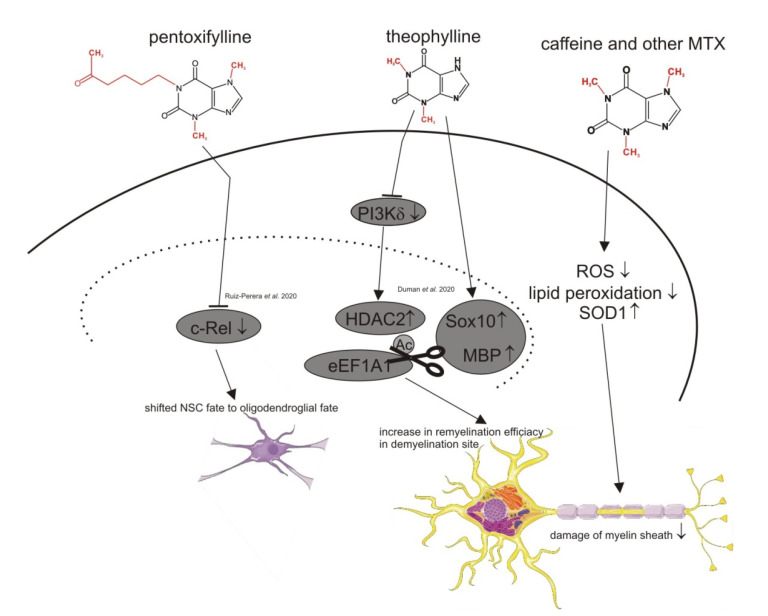
Summary of the molecular mechanisms reported in recent literature potentially mediating the beneficial effects of methylxanthines in respect to multiple sclerosis. Ac = Acetylation, eEF1A1 = eukaryotic translation elongation factor 1 alpha 1, HDAC2 = histone deacetylase 2, SOX10 = SRY-box transcription factor 10, MBP = myelin basic protein, ROS = reactive oxygen species, SOD1 = superoxide dismutase 1, c-Rel = proto-oncogene c-Rel, NSC = neuronal stem cells, PI3Kδ = phosphatidylinositol-3-kinase delta.

Although no clear association between methylxanthines and the risk for MS has been reported, animal studies with caffeine have shown an interesting therapeutical potential of methylxanthines in respect to MS, as summarized in Figure 3 and Table 6. One study demonstrated the neuroprotective effect of caffeine in experimental autoimmune encephalomyelitis (EAE) rats. An adenosine receptor mediated shift from Th1 to Th2 cell cytokine function revealed caffeine as a potential immunomodulatory drug [76]. A more recent study by Duman et al. discovered that theophylline enhanced CNS and PNS remyelination by increasing HDAC2, SOX10 and MBP protein levels in young adults and old mice after inducing a focal demyelinating lesion in the spinal cord. The increased remyelination efficiency of theophylline only occurred in the lesion site [77]. Ruiz-Perera et al. also discovered a potential link between methylxanthines and MS. In their study, the authors found that pentoxifylline completely shifted human neuronal stem cells to the oligodendroglial fate by inhibiting the c-REL pathway [78].

## 5. Conclusions

In summary, the recent literature provides further evidence for beneficial effects of methylated xanthine-derivatives like caffeine, theobromine, theophylline, pentoxifylline and propentofylline on pathological processes leading to neurodegenerative diseases, including Alzheimer´s disease, Parkinson´s disease and multiple sclerosis. However, recent clinical trials show a clearer association between methylxanthine and PD than AD or MS. Nevertheless, in respect to AD, several recent experimental studies have suggested molecular mechanisms by which methylxanthines could mediate protective effects. Further studies are needed to clarify the suggested relationship between methylated xanthine variants and the pathomechanisms present in MS in more detail. 

The physiological plasma levels of caffeine (and its naturally occurring metabolites) after daily intake of moderate amounts of coffee are not sufficient to trigger all the observed effects and higher levels that can be reached by pharmaceutical intake. It can also have unintended side effects like tremors, palpitations and arrhythmias, therefore the use of synthetic xanthine derivatives becomes more and more important. Regarding PD, the caffeine analogue istradefylline has potential to be an application of a synthetic methylxanthine for the treatment of a neurodegenerative disorder. Moreover, it is currently under investigation for the treatment of AD. These findings show that examining and synthesizing such xanthine derivatives, and moreover analyzing them in clinical trials, could be a promising approach in therapies of neurodegenerative diseases.

## Figures and Tables

**Table 1:** Summary of recent clinical studies investigating the relationship between methylxanthines and Alzheimer´s disease (AD). *n*: sample size; CSF: cerebrospinal fluid; CRP: c-reactive protein.

Author	Year	Type of Study/*n*	Substance	Outcome
Kim et al. [7]	2019	clinical trial/ 411 healthy participants (142 coffee drinkers and 269 reference participants)	coffee	association of coffee intake with reduced amyloid deposition
Larsson and Orsini [10]	2018	dose-response meta-analysis/ eight observational prospective studies	coffee	no evidence for a relationship between coffee-consumption and risk of dementia or AD
Iranpour et al. [14]	2019	clinical trial/ 1440 participants	caffeine	weak positive relation of high caffeine intake with cognitive function
Dong et al. [17]	2020	clinical trial/ 2500 participants	caffeine	significant associations with cognitive performance for coffee, caffeinated coffee and caffeine from coffee, but not for decaffeinated coffee
Leeuw et al. [20]	2020	longitudinal study/ 299 participants	theobromine	high levels of theobromine detected in CSF are associated with clinical progression to dementia
Sanders et al. [6]	2020	systematic review of clinical trials, epidemiology and meta-analyses	propentofylline	propentofylline as phosphodiesterase inhibitor showing improvement of cognition and dementia severity in mild-to-moderate AD
Moua et al. [24]	2020	systematic review and meta-analysis/11 studies and 61,047 participants	coffee	significant association between coffee and CRP levels when analyzing the three studies with the largest sample size but no significant association when combining all studies
Rodas et al. [25]	2020	clinical trial/244 participants	caffeine	regular caffeine consumption induced very limited anti-inflammatory effects

**Table 2:** Summary of recent animal and cell culture studies investigating the relationship between methylxanthines and Alzheimer´s disease (AD). FAD: familial Alzheimer´s disease. A1AR: adenosine A1 receptor. A2AR: adenosine A2 receptor. LPS: lipopolysaccharide.

Author	Year	Used Model	Substance	Outcome
Liang Jin et al. [27]	2020	APP/PS1 mice	caffeine	intestinal permeability and oral absorption were not affected in the FAD mouse model
Zappettini et al. [28]	2019	THY-Tau22 mice	caffeine	consumption during pregnancy accelerates the development of cognitive deficits in offspring in a model of tauopathy
Yoneda et al. [30]	2017	C57BL/6NCr mice	theobromine	up-regulated cerebral brain-derived neurotrophic factor and facilitated motor learning
Orr et al. [31]	2018	mice with AD-like amyloid plaque pathology	istradefylline	reduced memory deficits
Franco et al. [32]	2020	primary cultures of neurons and microglia from control and APP_Sw,Ind_ mice	antagonists of A2AR	high levels of theobromine detected in CSF are associated with clinical progression to dementia
Gastaldo et al. [33]	2020	synthetic brain membranes	caffeine	caffeine is able to affect Aβ peptide aggregation in AD through a membrane-mediated pathway
Gupta et al. [35]	2019	in silico study	caffeine	disorganization of cross-β structures of Aβ17-42 fibrils in the presence of caffeine
Janitschke et al. [21]	2019	SH-SY5Y cells	caffeine, theobromine, theophylline, pentoxifylline, propentofylline	MTX reduce Aβ levels via pleiotropic molecular mechanisms and decrease oxidative stress, cholesterol levels and Aβ aggregation
Fabiani et al. [36]	2018	AchR-rich membrane fragments from *T*. *californica* and HEK293 cells	caffeine	pharmacological activity of caffeine in the cholinergic system
Kumar et al. [37]	2019	primary hippocampal neurons	caffeine	AchE inhibitory potential, improved neuronal survival and protection from neurodegeneration
Badshah et al. [41]	2019	LPS-injected mouse model	caffeine	prevention of LPS-induced oxidative stress and suppression of inflammatory mediators
Khan et al. [42]	2019	HT-22 and BV-2 cells, B57BL/6N mice	caffeine	modulation of cadmium-induced oxidative stress, neuroinflammation, and cognitive impairments by regulating nrf-2/ho-1 in vivo and in vitro
Zhao et al. [45]	2020	HEK293 cells	caffeine	inhibition of notum activity by binding at the catalytic pocket
Nabbi-Schroeter et al. [48]	2018	Sprague-Dawley rats	caffeine	no long-persistent upregulation of functionally available A1Ars under their conditions
Mendiola-Precoma et al. [49]	2017	rat brain AD model	theobromine	theobromine-induced changes in *A1AR* expression and distribution
Ciaramelli et al. [51]	2021	SH-SY5Y cells	theobromine	theobromine hinders Aβ peptide aggregation and toxicity
Janitschke et al. [52]	2020	SH-SY5Y cells	caffeine, theobromine, theophylline, pentoxifylline, propentofylline	different or inverse transcriptional regulatory effects of caffeine compared to the other tested MTX on AD-related genes

**Table 3:** Summary of recent clinical studies investigating the relationship between methylxanthines and Parkinson´s Disease (PD). *n*: sample size; CSF: cerebrospinal fluid.

Author	Year	Type of Study/*n*	Substance	Outcome
Bakshi et al. [59]	2020	cross sectional, case-control/197 healthy control vs. 369 idiopathic PD patients	caffeine, urate	the authors found a robust inverse association between idiopathic PD and caffeine intake and urate plasma levels
Fujimaki et al. [60]	2018	clinical trial/31 healthy control vs. 108 PD patients without dementia^1^	caffeine, theophylline, theobromine, paraxanthine and other downstream metabolites	absolute lower levels of caffeine and metabolites were found to be a promising biomarker for early PD
Crotty et al. [62]	2020	clinical trial/samples from “23andMe” study, LRRK2 longitudinal study and LRRK2 cross-sectional study (*n* = 380)	caffeine, theophylline, paraxanthine and other downstream metabolites, trigonelline (non-xanthine constituent of coffee)	significantly lower plasma and CSF levels of caffeine and downstream metabolites in PD patients, even more in LRRK2 mutation carriers
Ohmichi et al. [63]	2018	clinical trial/31 PD patients vs. 33 age-matched controls	theophylline	theophylline serum levels were significantly lower in PD patients compared to control
Hong et al. [64]	2020	meta-analysis/13 studies (9 healthy cohort, 4 PD cohort)	caffeine	caffeine consumption resulted in a significantly lower rate of PD
Maclagan et al. [65]	2020	computational & pharmacoepidemiologic study ranking 620 drugs, case-control study/14,866 PD and 74,330 controls	pentoxifylline, theophylline, dexamethasone	the authors state, that corticosteroids and the found methylxanthines should be investigated as disease-modifying drugs
Sako et al. [67]	2017	meta-analysis/six studies	istradefylline	20 and 40 mg/day of istradefylline revealed significantly decreased durations of “off episodes” in PD patients

**Table 4:** Summary of recent animal and cell culture studies investigating the relationship between methylxanthines and Parkinson´s disease (PD).

Author	Year	Used Model	Substance	Outcome
Luan et al. [69]	2018	Injected α-synuclein fibrils intra-striatal in mice	caffeine	reduced inclusion of α-synuclein, apoptosis, microglial activation and astrogliosis after caffeine treatment
Khadrawy et al. [70]	2017	rotenenoe induced PD mice model	caffeine	recovering dopamine levels in midbrain and striatum ameliorating motor symptoms, antioxidative and anti-inflammatory effect of caffeine, prevention of neurodegeneration through less lewy bodies
Pardo et al. [71]	2020	pimozide induced PD mice model	theophylline	reversed locomotion, catalepsy and tremulous jaw movement
Rohilla et al. [72]	2019	perphenazine induced catatonia in rats	newly synthesized xanthine derivatives	most xanthines significantly lowered catatonia score, most potent MTX shows a similar response as L-DOPA

**Table 5:** Summary of recent clinical studies investigating the relationship between methylxanthines and multiple sclerosis (MS). *n*: sample size.

Author	Year	Type of Study/n	Substance	Outcome
Lu et al. [75]	2020	mendelian randomization study/14,802 MS subjects vs. 26,703 healthy controls	coffee	The authors state that coffee consumption and the risk of MS might not be causally associated

**Table 6:** Summary of recent animal and cell culture studies investigating the relationship between methylxanthines and Multiple Sclerosis (MS).

Author	Year	Used Model	Substance	Outcome
Duman et al. [77]	2020	lysolecithin induced demyelination lesion in the spinal cord of mice	theophylline	increased remyelination efficiency within lesion side via through increase of HADC2, SOX10 and MBP protein levels
Ruiz-Perera et al. [78]	2020	human neuronal stem cells	pentoxifylline	through inhibition of the c-REL pathway pentoxifylline shifted stem cell differentiation to oligodendrioglial cells
